# Identification of gene signatures and potential pharmaceutical candidates linked to COVID-19-related depression based on gene expression profiles

**DOI:** 10.3389/fphar.2025.1562774

**Published:** 2025-08-22

**Authors:** Shaojun Chen, Yiyuan Luo, Lihua Zhang

**Affiliations:** ^1^ Department of Traditional Chinese Medicine, Zhejiang Pharmaceutical University, Ningbo, China; ^2^ School of Health Management, Zhejiang Pharmaceutical University, Ningbo, China

**Keywords:** COVID-19, gene-expression signature, machine learning, major depressive disorder, trichostatin A

## Abstract

**Background:**

Acute and long-term mental health disorders correlate with coronavirus disease 2019 (COVID-19). The underlying mechanisms responsible for the coexistence of COVID-19 and depression remain unclear, and more research is needed to find hub genes and effective therapies. The main objective of this study was to evaluate gene-expression profiles and, identify key genes, and discovery potential therapeutic agents for co-occurrence in COVID-19 and major depressive disorder (MDD).

**Methods:**

Initially, we identified differentially expressed genes (DEGs) in datasets from COVID-19 (GSE188847) or MDD (GSE101521). Subsequently, we employed two machine learning analyses—least absolute shrinkage and selection operator (LASSO) and random forest algorithms– to pinpoint shared hub gene between the two diseases. Furthermore, the LINCS L1000 characteristic direction signatures search engine (L1000CDS2) was utilized for drug repurposing studies based on the gene-expression signatures. Finally, molecular dynamics (MD) simulations were conducted to investigate the binding interactions between molecules and the target proteins.

**Results:**

We uncovered 60 DEGs that overlapped between the two datasets but exhibited distinct patterns of expression in each dataset. Subsequent machine learning analyses revealed EMILIN3, OPA3, and TFCP2 as potential shared hub genes underlying both diseases. Furthermore, L1000CDS2 analysis indicated that trichostatin A (TSA), a metabolite derived from Streptomyces, could potentially reverse the altered gene expression. Molecular docking and molecular dynamics simulations revealed that complexes of TSA–perturbed protein spontaneously form and are highly stable.

**Conclusion:**

EMILIN3, OPA3, and TFCP2 are likely to be potential shared hub genes in both COVID-19 and depression. Meanwhile, TSA may serve as a therapeutic option for treating COVID-19-associated depression. Given the inherent constraints of computational modeling, further biological validation studies would help establish the significance of these preliminary findings.

## Introduction

Corona virus disease 2019 (COVID-19), which became pandemic at the beginning of 2020, caused immense health and social crises worldwide (Morawska et al., 2024). Currently, it is an endemic disease, remaining a matter of global concern, with persistent health implications (Cohen and Pulliam, 2023). As well as the direct viral effects of SARS-CoV-2 and people’s fear of infection, COVID-19 caused personal and societal disruptions including societal constraints, shutdowns, cessation of educational and commercial activities, livelihood disruption, economic upset, and so on (The Lancet Public, 2020; Moeti et al., 2022; Dregan and Armstrong, 2023). These direct and indirect effects were detrimental to people’s mental health, resulting in substantial and enduring human suffering (The Lancet Public, 2020; Collaborators, 2021; Kola et al., 2021; Moeti et al., 2022; Dregan and Armstrong, 2023; Lovik et al., 2023).

Depression is a common mental health issue that can happen to anyone, noted for its disabling effects and expense to individuals and healthcare systems (Organization, 2023; Marwaha et al., 2023). It can lead to difficulties in various facets of life, including social settings, family life, occupation, and education (Organization). Depression is distinguished by persistent low mood or a prolonged absence of enjoyment or interest in activities, and is also linked to a higher likelihood of suicide (Organization, 2023; Marwaha et al., 2023). It is a prominent global public health problem, and approximately 280 million people worldwide, about 3.8% of the total population, suffer from depression, with 5% of adults and 5.7% of adults over 60 years old being affected (Organization, 2023; Marwaha et al., 2023).

The COVID-19 pandemic markedly increased the risk of depression, particularly among older individuals who lack family support (Oh et al., 2023), as well as females and younger populations (Collaborators, 2021). An estimated extra 53.2 million cases of depression occurred worldwide in 2020 as a result of COVID-19, representing a 27.6% increase and an overall prevalence of 3,152.9 cases per 100,000 population (Collaborators, 2021). The results of questionnaires administered to young people in Germany who did not have pre-pandemic health issues showed that their health worsened considerably, with 61% reporting depression and 44% reporting anxiety symptoms in 2021 (Kleine et al., 2023). In Milan, Italy, 35.8% of 226 COVID-19 survivors self-reported experiencing psychopathological symptoms, including persistent depressive traits, 3 months after hospital discharge (Mazza et al., 2021). Nevertheless, the genetic foundation underlying the comorbidity between COVID-19 and depression comorbidity requires further investigation.

**FIGURE 1:**
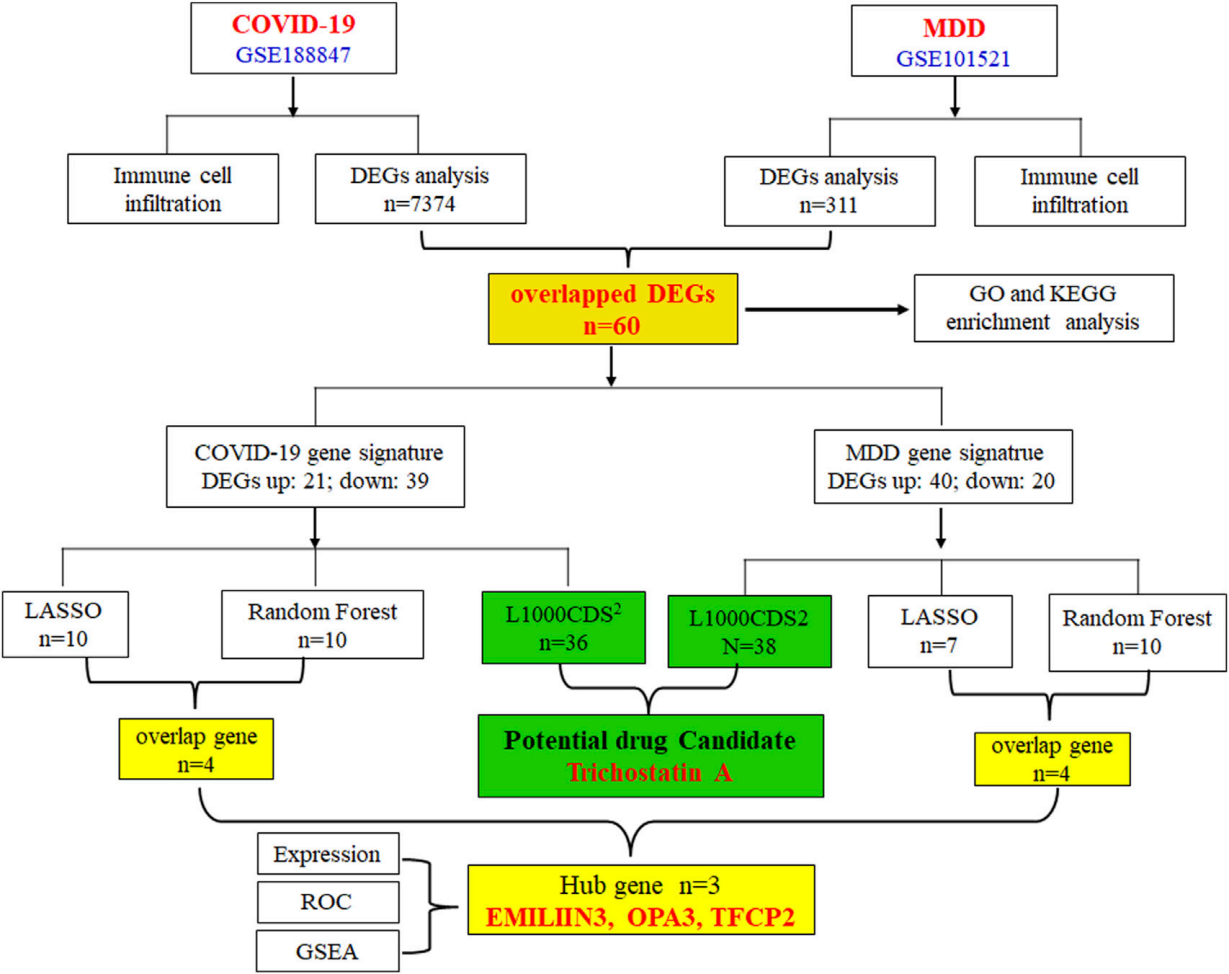
A flowchart of the experimental procedures used in this study.

Therefore, the primary objective of this research was to utilize extensive genetic data to enhance our comprehension of the pathological mechanisms underlying COVID-19 depression, and to propose potential therapeutic compounds for its treatment. The gene-expression datasets for COVID-19 and major depression disorder (MDD) were sourced from the publicly available Gene Expression Omnibus (GEO) database. Through the application of various bioinformatics tools, we thoroughly analyzed these datasets to identify differentially expressed genes (DEGs), and employed machine learning techniques to identify pivotal genetic markers. Furthermore, we searched for small-molecule compounds that were predicted to regulate the abnormal genetic alterations associated with COVID-19-related depression. The workflow of this study is depicted in Figure 1.

## Materials and methods

### Data information and processing

Gene expression data pertaining to patients were retrieved from the GEO database (https://www.ncbi.nlm.nih.gov/geo/). The GSE188847 dataset aimed to identify numerous cognition-associated long noncoding RNAs that were differentially expressed in brain tissue from patients with severe COVID-19 (Lee et al., 2024). This dataset comprised 20 COVID-19 samples and 22 uninfected controls, all sourced from the frontal cortex of postmortem brain tissue. Furthermore, the GSE101521 dataset profiled whole-transcriptome expression and exon usage in brain tissue from individuals diagnosed with MDD (Pantazatos et al., 2017). This dataset encompassed 29 non-psychiatric controls and 9 MDD patients who died suddenly but did not die by suicide (Pantazatos et al., 2017). All subjects were intentionally selected due to their sudden deaths to minimize metabolic disturbances arising from agonal effects, and each brain specimen underwent rigorous screening to confirm the absence of gross neuropathology and negative toxicology results for psychotropic, illicit psychoactive, and neurotoxic drugs (Pantazatos et al., 2017). These samples were obtained from the dorsal lateral prefrontal cortex. Due to the stringent selection criteria, large sample sizes or similar dataset remains challenging. Both the original studies involved human specimens and were approved by their respective ethics review boards.

### DEG identification and enrichment analysis

The Limma package of R software, which is integrated into GEO2R, was employed to identify DEGs in the GSE188847 dataset by comparing control samples with those from COVID-19 patients. Similarly, the Limma package was used to detect DEGs in the GSE101521 dataset by comparing control samples with those from MDD patients. The selection of DEGs was determined according to the criteria of |FoldChange|>0 and adj. *P* < 0.05. Following DEG identification, a range of analyses were performed on the selected DEGs utilizing online web tools (https://bioinformatics.com.cn/). These analyses included generating a volcano plot, gene ontology (GO) enrichment analysis, and Kyoto Encyclopedia of Genes and Genomes (KEGG) enrichment analysis.

### Machine learning-based selection of hub genes

Machine learning methods hold immense potential to identify pivotal features, such as biomarkers, within biomedical applications (Li and Sillanpaa, 2012; Wang et al., 2016). Both the least absolute shrinkage and selection operator (LASSO) and random forest (RF) algorithms are well-established machine learning methods. LASSO regression analysis was carried out using the glmnet package in R, and the RF algorithm was carried out using the randomForest package to identify hub genes among DEGs related to COVID-19 and MDD.

The hub genes were ultimately determined by intersecting the results from LASSO and RF assessments, with the overlapping genes designated as those potentially associated with COVID-19-related depression. The relative expressions and area under the ROC curve (AUC) of these hub genes were calculated using the GSE188847 and GSE101521 raw datasets.

### Single-gene gene set enrichment analysis (GSEA)

We used GSEA to explore the potential functions of signature genes linked to COVID-19-related depression. Initially, the samples from the GSE188847, comprising those of COVID-19 patients and unaffected individuals, as well as the MDD and non-psychiatric control samples from GSE101521 datasets, were categorized into low-expression and high-expression groups based on the expression levels of each signature gene. Subsequently, the official GSEA4.3.3 software was used to identify specific pathways associated with these two groups. Specifically, the GSE188847 enrichment analysis centered on the reactome–SARS-CoV-2 infection pathway and the GSE101582 enrichment analysis focused on the HP–depression pathway.

### Immune infiltration analysis

CIBERSORT is a deconvolution algorithm that is used to quantify cell types and evaluate the distribution of 22 immune cells in intricate tissues by analyzing their gene expression profiles (Newman et al., 2015). In our study, we employed the CIBERSORT algorithm to comprehensively analyze the immune infiltration patterns within the COVID-19 (GSE188847) and MDD (GSE101521) datasets.

### Potential drug repurposing based on gene signatures

The LINCS L1000 library is a large-scale resource containing data on 978 genes that act as genome-wide markers capable of inferring the expression levels of 81% of non-measured transcripts (Subramanian et al., 2017). This cost-effective and high-throughput methodology holds substantial value in drug repurposing studies (Subramanian et al., 2017). L1000CDS^2^ is a useful tool in drug discovery, facilitating rapid searches through the L1000 dataset to discover and rank small molecules that either reverse or mimic user-provided gene expression signatures (Duan et al., 2016).

To discover potential therapeutic agents for alleviating COVID-19-related depression, we submitted the details of upregulated and downregulated DEGs independently derived from the COVID-19 (GSE188847) and MDD (GSE101521) datasets to the L1000CDS2 search engine. Subsequently, the top 50 drug perturbations that might reverse the unique gene expression signatures associated with either COVID-19 or MDD were obtained. The drugs that overlap in both lists are potential candidates for treating both COVID-19 and MDD.

### Docking and molecular dynamics (MD) simulations

Trichostatin A (TSA), identified as a promising drug candidate of interest from our L1000CDS^2^ results, along with its associated perturbed proteins, were further investigated. The chemical structure of TSA (CID: 6376322) was retrieved from the PubChem database, and processed into a pdbqt file using MGLTools 1.5.7. Furthermore, the crystalline structures of the perturbed proteins were obtained from the PDB database (https://www.rcsb.org/), and redundant structures such as small molecules and water were removed. AutoDock Vina 1.1.2 was employed to dock TSA with the perturbed proteins. Based on the docking results, conformations with higher binding affinity and binding within the protein surface pocket were selected for MD simulations.

MD simulations (100 ns) were performed utilizing the Gromacs 2022 program. The procedures and parameter settings of the MD simulations were similar to those described in a previously published study (Wang X. et al., 2024). Briefly, the small molecule was modeled using the GAFF force field, proteins were modeled using the AMBER14SB force field, and water molecules were modeled using the TIP3P water model. MD simulations were performed under constant temperature and pressure, along with periodic boundary conditions. During the MD simulations, all relevant hydrogen bonds were confined using the LINCS algorithm with an integration time of 2 fs. Electrostatic interactions were computed using the particle-mesh Ewald method, with a cutoff distance of 1.2 nm. Additionally, the cutoff distance for non-bonded interactions was set at 1 nm and updated every 10 steps. The V-rescale thermostat was used to maintain the simulation temperature at 298 K and the Berendsen barostat controlled the pressure at 1 bar. At 298 K, 100 ps NVT and NPT equilibration simulations were conducted, followed by a 100 ns MD simulation of the TSA–perturbed protein complexes. Configurations were saved every 10 ps. After MD simulations were complete, the free energy of ligand binding was calculated using MM/GBSA methods with the g_mmpbsa program, and the MD trajectories were analyzed using VMD and PyMOL software.

## Results

### Identification of DEGs

**FIGURE 2:**
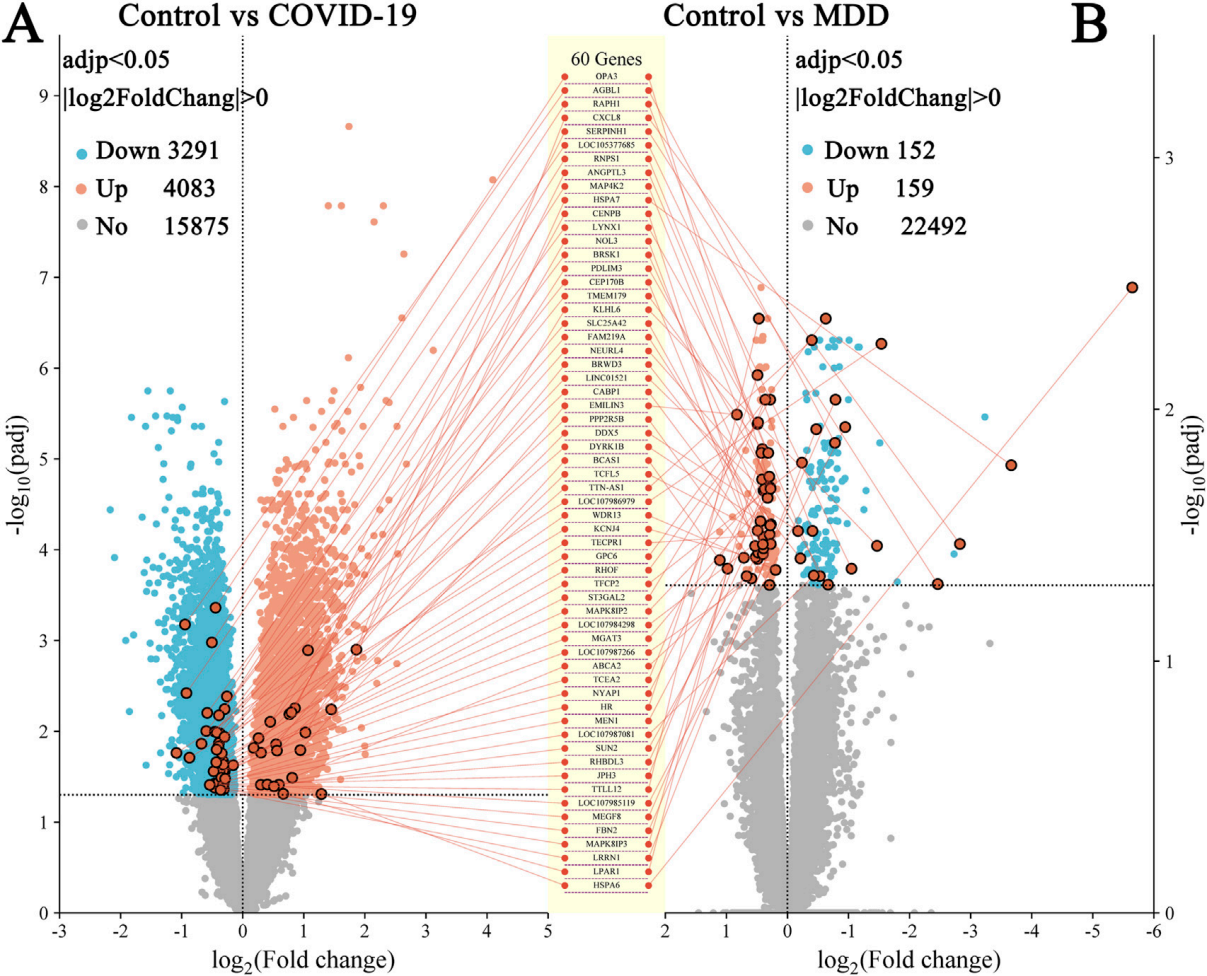
Volcano plots of DEGs. **(A)** GSE188847; **(B)** GSE101521. There were 60 DEGs common between the two datasets.

In the GSE188487 dataset associated with COVID-19, 7,374 DEGs (p < 0.05) were detected. These included 4,083 upregulated genes and 3,191 downregulated genes (Figure 2A; Supplementary Table S1). In addition, the GSE101521 dataset related to MDD revealed 311 DEGs (p < 0.05), with 159 upregulated and 152 downregulated genes (Figure 2B; Supplementary Table S2).

**FIGURE 3:**
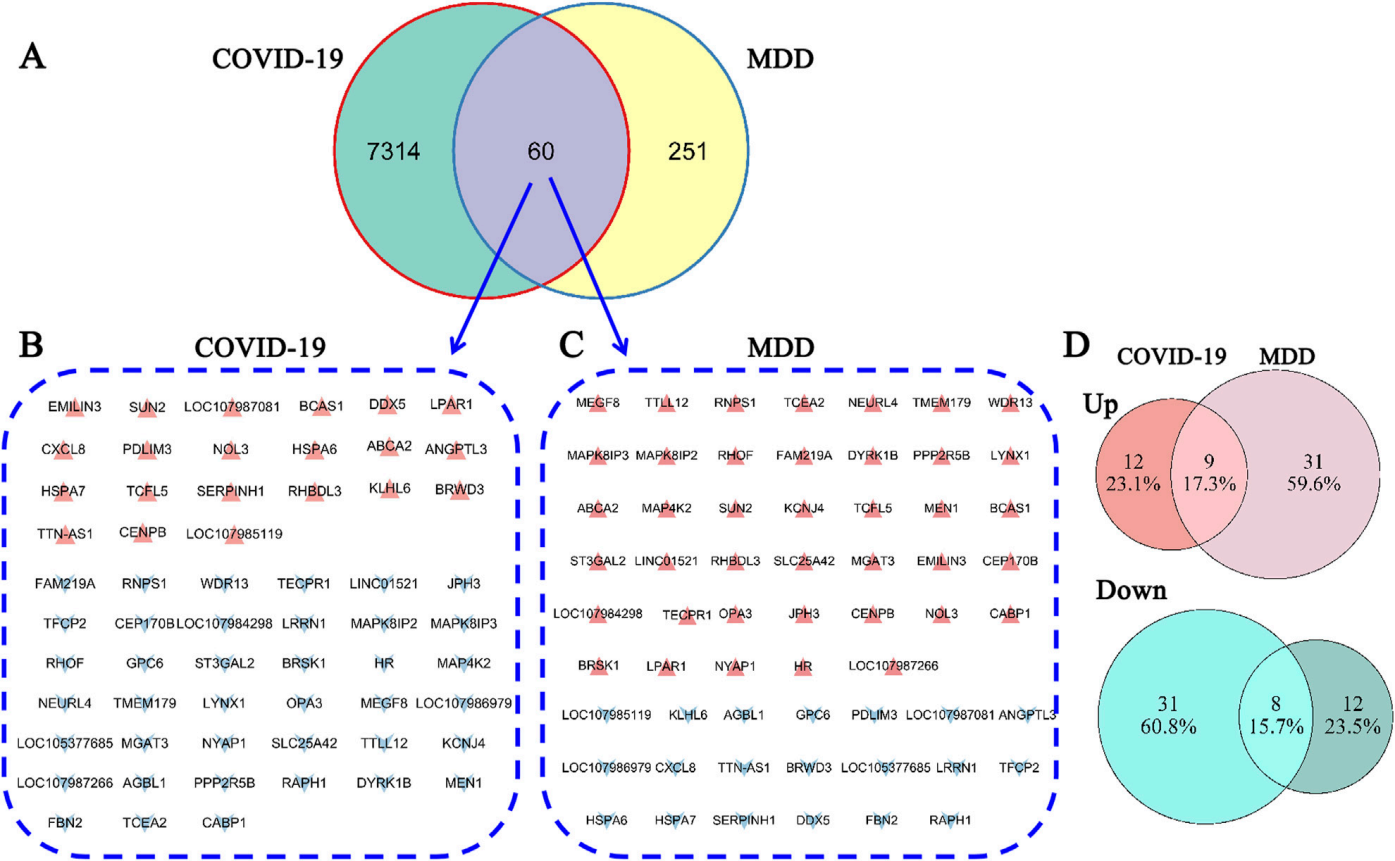
The gene expression signatures for the two datasets. **(A)** The overlapping genes; **(B)** the gene signature for COVID-19; **(C)** the gene signature for MDD; **(D)** the overlap of upregulated and downregulated DEGs.

DEGs that overlapped between both datasets identified 60 common DEGs (Figures 2, 3A; Supplementary Table S3). This finding suggested that the underlying processes associated with depression and COVID-19 converge. Moreover, the common DEGs had different expression patterns in the COVID-19 (GSE188847) and MDD (GSE101521) datasets (Figures 3B,C). Among the 60 overlapping DEGs, the COVID-19 dataset comprised 21 upregulated genes and 39 downregulated genes, whereas the MDD dataset contained 40 upregulated genes and 20 downregulated genes (Figure 3D). This result suggests that these common genes have distinct physiological roles in the two pathological conditions.

### Gene ontology enrichment and KEGG enrichment analysis

**FIGURE 4:**
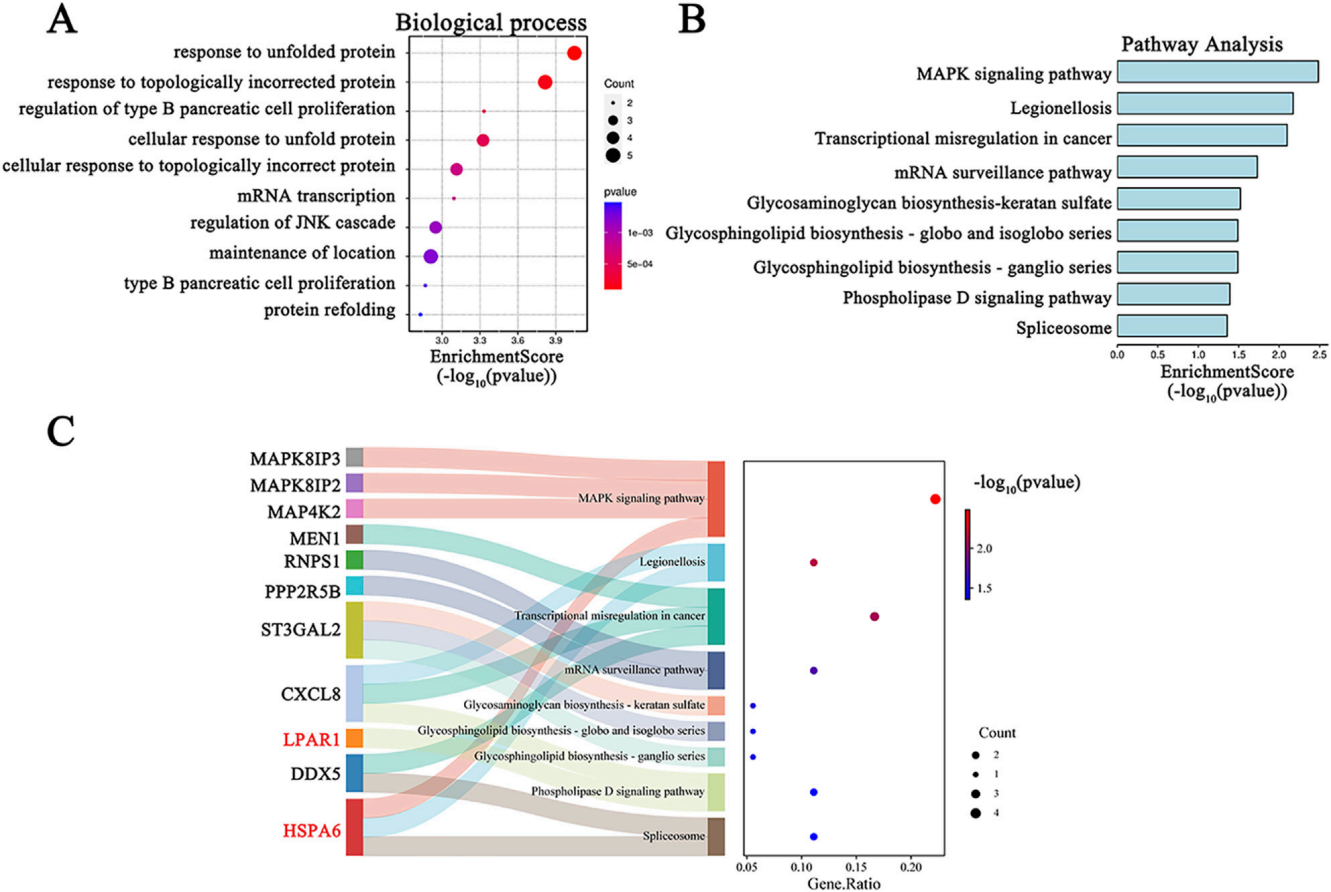
GO and KEGG functional enrichment analysis for DEGs in COVID-19-related depression. **(A)** GO biological process enrichment analysis; **(B)** KEGG pathway enrichment analysis; **(C)** the relationship between hub genes and KEGG pathways depicted by a Sankey diagram.

Functional enrichment analyses were conducted to clarify the underlying biological functions of the 60 common DEGs. The top 10 biological processes are visualized in Figure 4A. The biological process terms of these genes were related to “response to unfolded protein”, “response to topologically incorrect protein”, and “regulation of type B pancreatic cell proliferation”. In addition, the KEGG pathway of these common DEGs included “MAPK signaling pathway”, “legionellosis”, and “Transcriptional misregulation in cancer” (Figure 4B). The Sankey diagram depicts the primary gene distributions in the different KEGG pathways (Figure 4C).

### Screening of hub genes via machine learning

**FIGURE 5:**
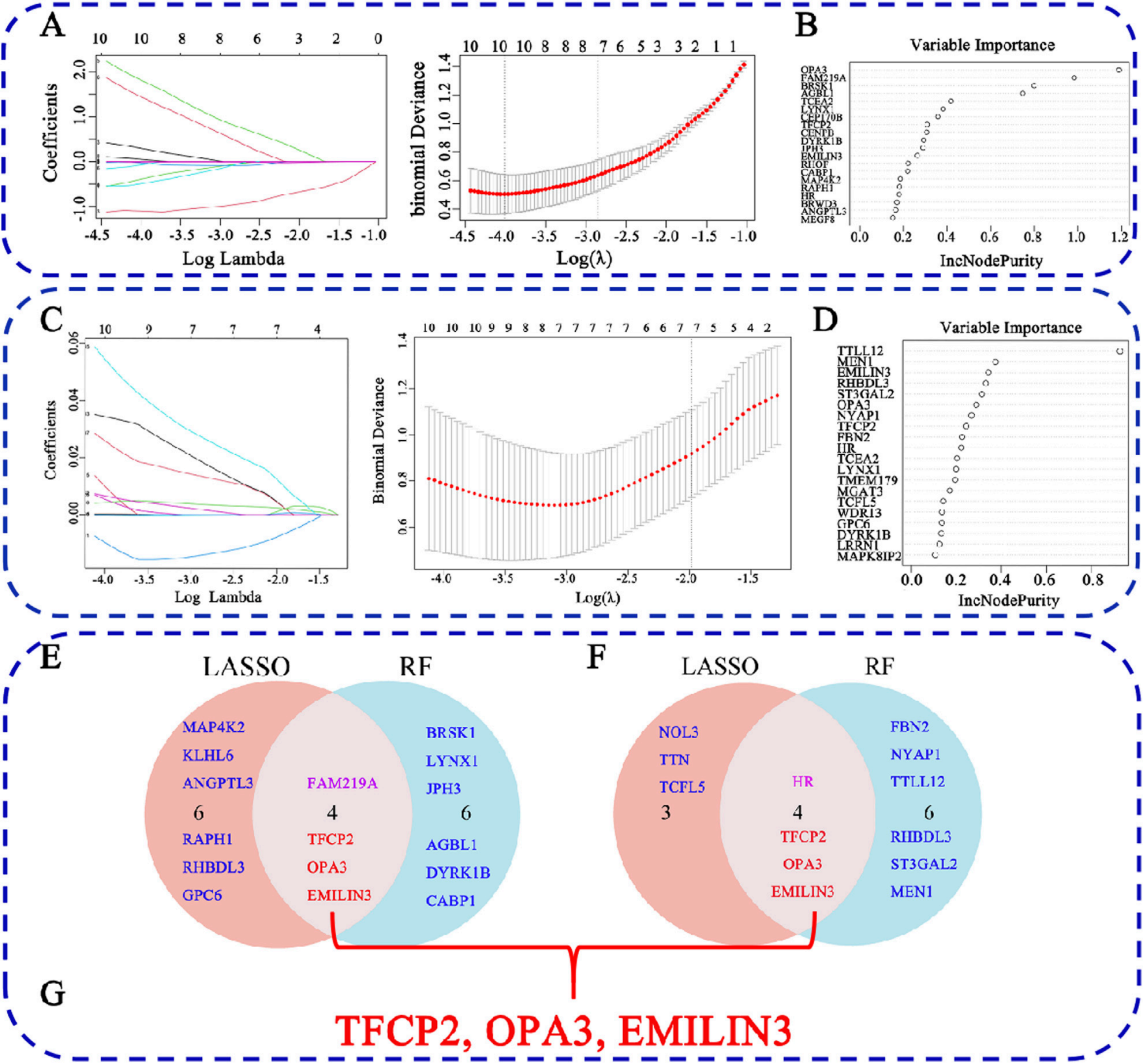
Identification of hub genes for COVID-19-related depression. **(A)** LASSO regression analysis for the COVID-19 dataset; **(B)** RF analysis for COVID-19; **(C)** LASSO regression analysis for the MDD dataset; **(D)** RF analysis for COVID-19; **(E)** the overlap of LASSO and RF results for COVID-19; **(F)** the overlap of LASSO and RF results for MDD; **(G)** the overlap hub gene for COVID-19 depression.

To identify characteristic genes from the 60 common DEGs associated with COVID-19 and MDD, two machine learning algorithms, LASSO regression and RF, were employed. For the COVID-19-related DEGs, LASSO regression identified 10 hub genes (Figure 5A), while RF also identified 10 hub genes (Figure 5B). Notably, several genes overlapped between the two methods, specifically transcription factor CP2 (TFCP2), family with sequence similarity 219 member A (FAM219A), outer mitochondrial membrane lipid metabolism regulator OPA3 (OPA3), and elastin microfibril interfacer 3 (EMILIN3) (Figure 5E).

In the context of MDD, LASSO regression identified seven hub genes (Figure 5C), and RF identified 10 hub genes (Figure 5D). Among these, four overlapped between both methods: HR lysine demethylase and nuclear receptor corepressor (HR), TFCP2, OPA3, and EMILIN3 (Figure 5F). Furthermore, when comparing COVID-19- and MDD-related hub genes, we found that three genes, i.e., TFCP2, OPA3, and EMILIN3, overlapped (Figure 5G). These three genes may represent hubs that are critical for understanding the link between COVID-19 and MDD.

**FIGURE 6:**
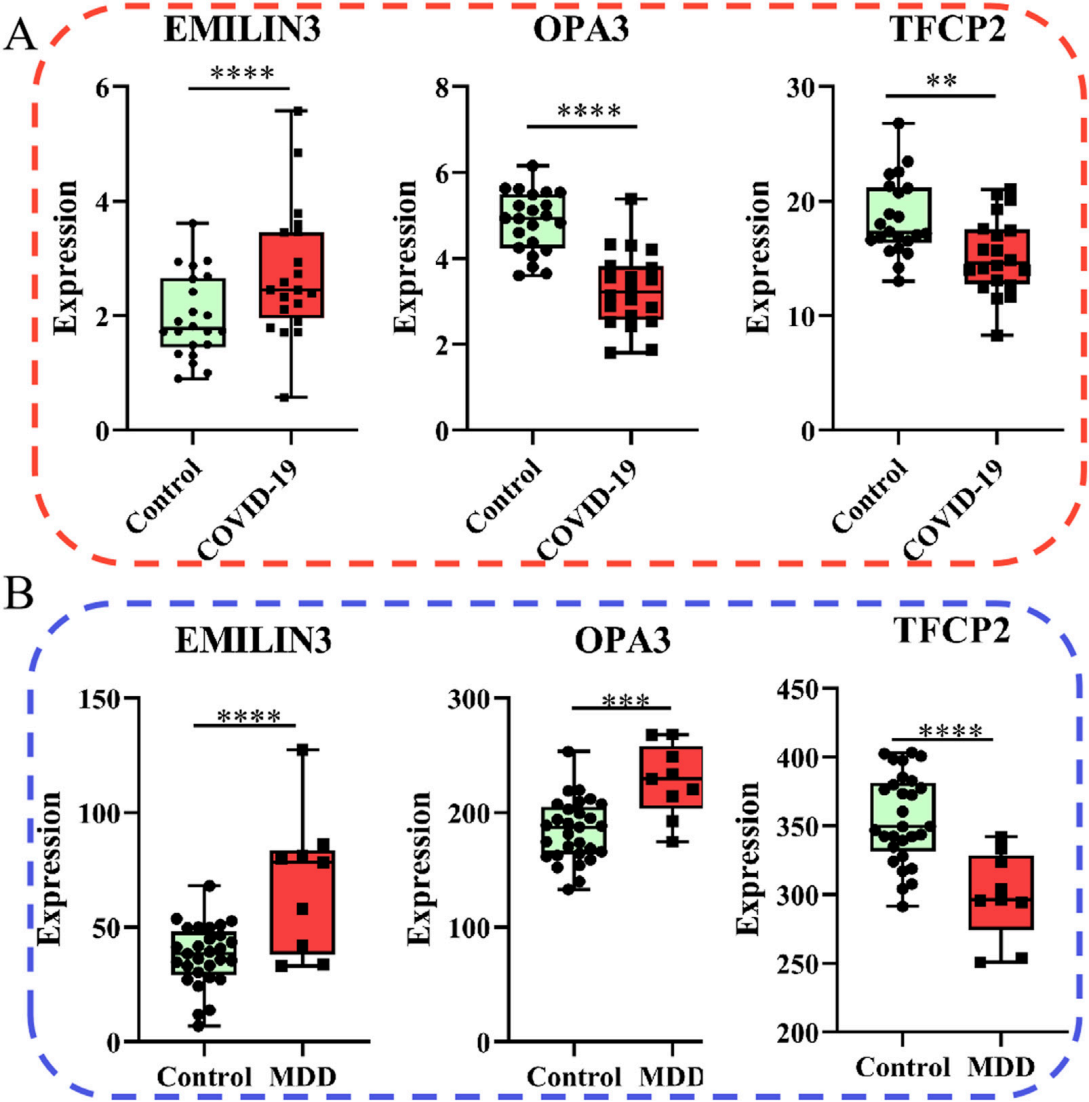
Relative expression of the three hub genes. **(A)** The COVID-19 dataset; **(B)** the MDD dataset.

Of note, the expression of EMILIN3 was highly upregulated in both the COVID-19 and MDD datasets (Figures 6A,B). Conversely, TFCP2 expression levels were notably down-regulated in both the COVID-19 and MDD datasets (Figures 6A,B). However, OPA3 was down-regulated in the COVID-19 dataset and up-regulated in the MDD dataset (Figures 6A,B). The different expression patterns of these core genes illustrate that gene-expression signatures can be different in two diseases, even if they share common genes.

**FIGURE 7:**
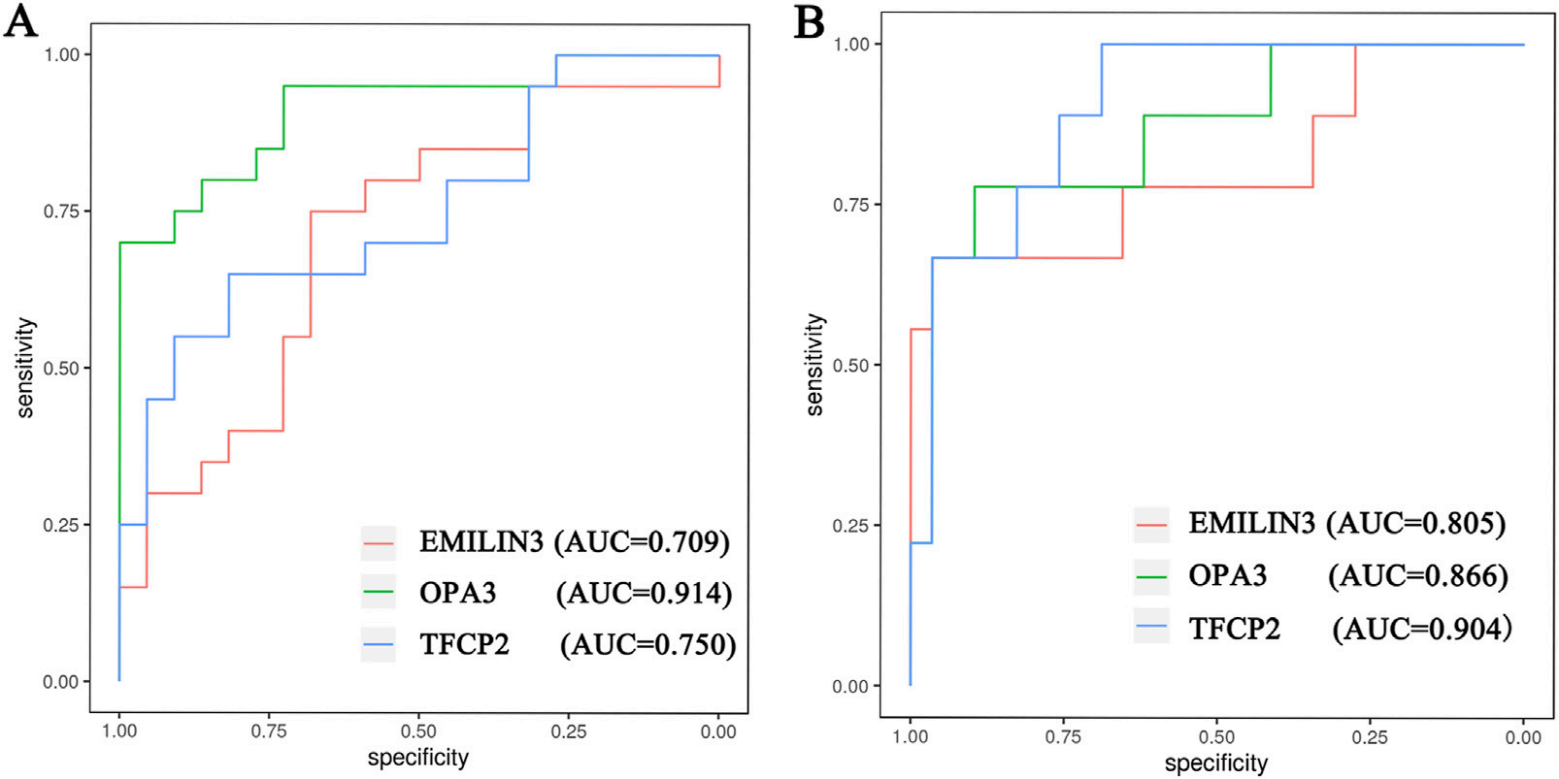
The ROC curve of the hub genes. **(A)** The COVID-19 dataset; **(B)** the MDD dataset.

Furthermore, the ROC curves demonstrated that the three hub genes exhibited ideal diagnostic efficiency (Figure 7). In the GSE188847 dataset, the AUC values for EMILIN3, OPA3, and TFCP2 were 0.709, 0.914, and 0.750, respectively (Figure 7A). In the GSE101521 dataset, the AUCs for EMILIN3, OPA3, and TFCP2 were 0.805, 0.866, and 0.904, respectively (Figure 7B). Notably, all these AUC values exceeded 0.7, indicating that these three genes performed well for diagnostic efficiency in both the COVID-19 dataset and in MDD dataset (Figure 7).

**FIGURE 8:**
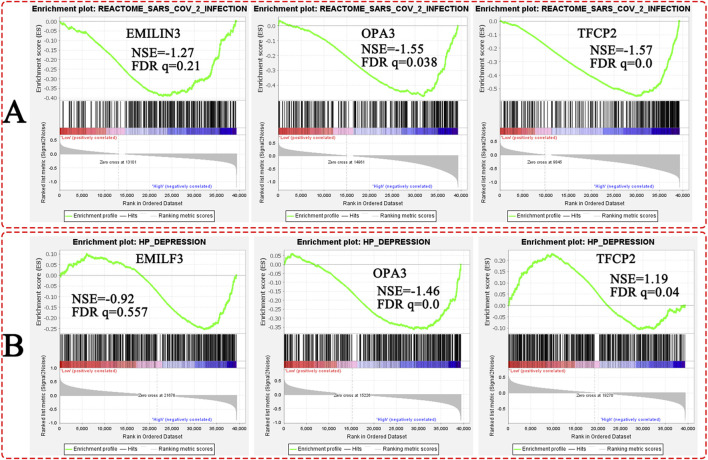
The single-gene GSEA of the three hub genes. **(A)** The COVID-19 dataset; **(B)** the MDD dataset.

The single-gene GSEA method was employed to dissect the relationships between the hub genes and a specific pathway. In the GSE188847 dataset, the three hub genes were observed, and had a similar pattern, in the reactome–SARS-CoV-2 infection pathway (Figure 8A). Furthermore, in the GSE101521 dataset, OPA3 and TFCP2 genes, but not EMILIN3, were found in the HP–depression enrichment pathway (Figure 8B).

### Assessment of immune cell infiltration

**FIGURE 9:**
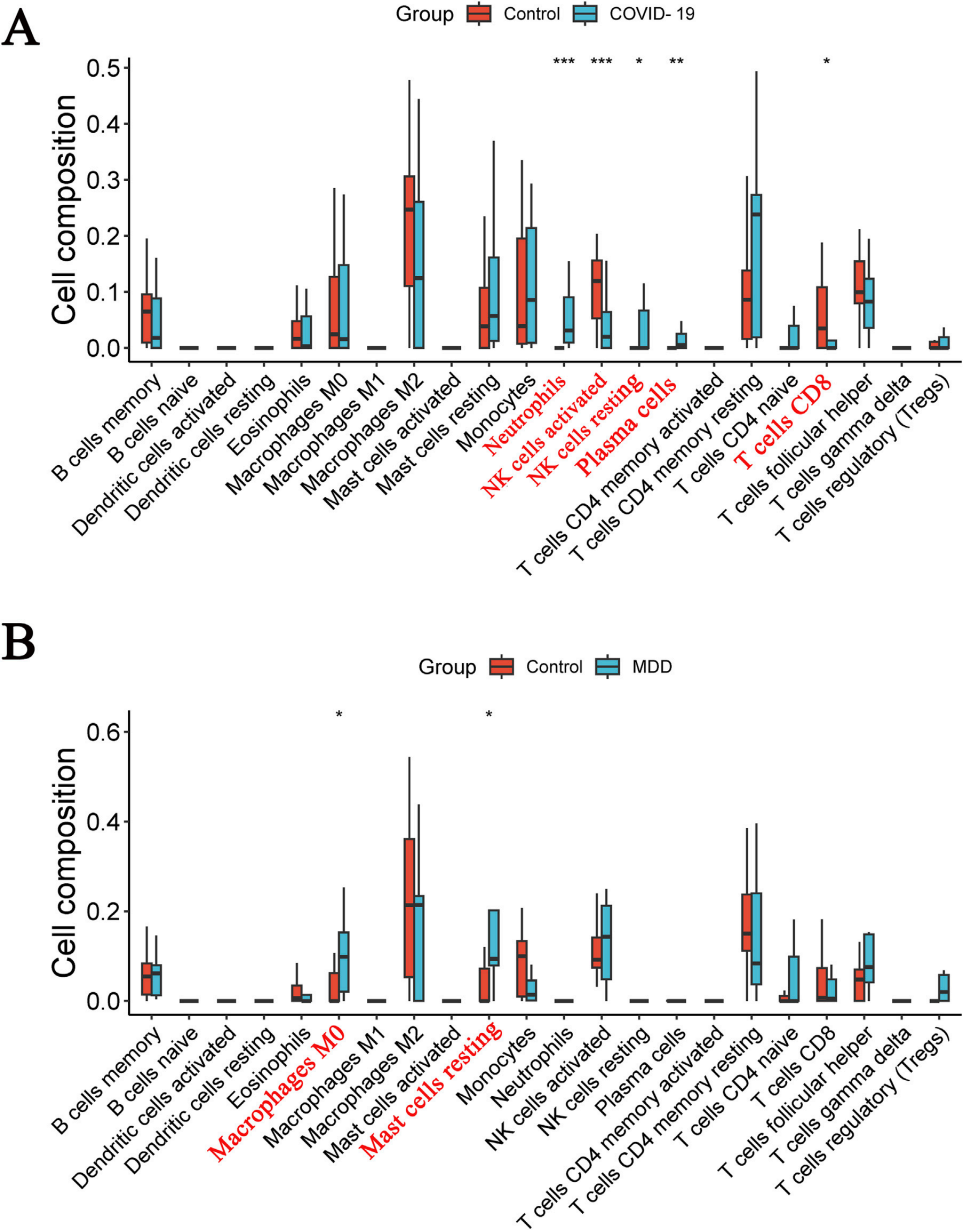
Diverse types of immune cells in patients. **(A)** The COVID-19 dataset; **(B)** the MDD dataset.

The CIBERSOFT algorithm was used to investigate immune cell infiltration in samples from patients with COVID-19 or MDD. As depicted in immune-cell maps (Figure 9), the two diseases displayed distinct patterns of immune cell infiltration. The most predominant cell types in COVID-19 patients were neutrophils, activated natural killer (NK) cells, resting NK cells, plasma cells, and CD8 T cells (Figure 9A). In MDD patients the most common cell types were M0 macrophages and resting mast cells (Figure 9B).

### Identification of small-molecule therapeutics based on gene signatures

**FIGURE 10:**
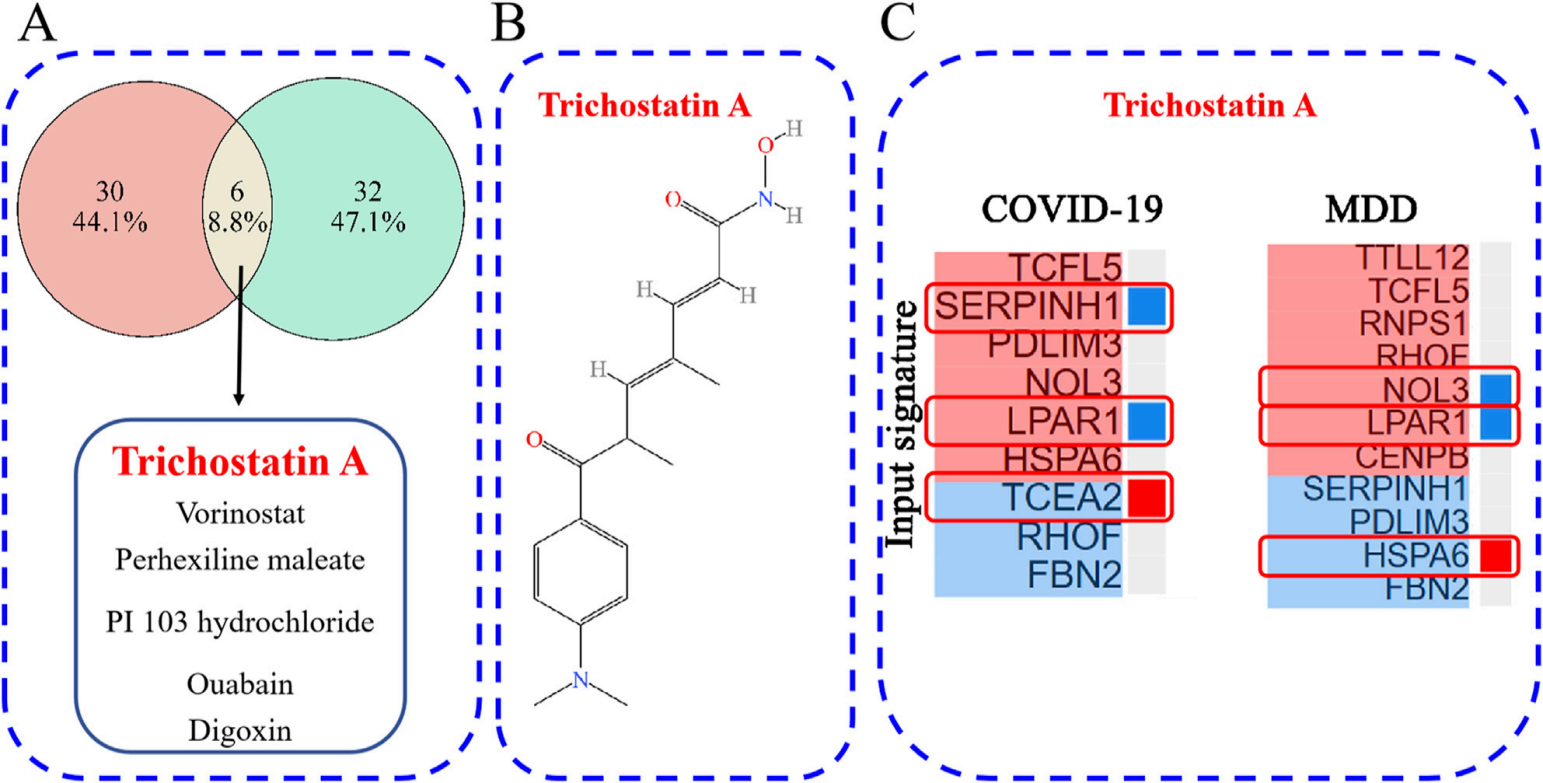
**(A)** The overlap of potential drug candidates for COVID-19-related depression; **(B)** the chemical structure of TSA; **(C)** genes that overlap between those perturbed by TSA and the provided input gene signature.

The L1000CDS^2^ web-based tool was used to predict small molecules capable of reversing the perturbed gene signature (up- and down-regulated DEGs). For the GSE188847 dataset, the top 50 small molecules predicted to target the COVID-19 gene signature, of which 36 were unique to this dataset, are shown in Supplementary Table S4 and Figure 10A. For the GSE101521 dataset, the top 50 chemical compounds predicated to target the MDD gene signature, with 38 unique to this dataset, are shown in Supplementary Table S5 and Figure 10A. Notably, six of these small-molecule compounds, namely, trichostatin A (TSA), vorinostat, perhexiline maleate, PI103 hydrochloride, ouabain, and digoxin were predicted to target both datasets (Figure 10A). These compounds may exert effects on both COVID-19 and MDD.

TSA has been reported to have therapeutic effects on both COVID-19 and depression (Chen et al., 2019; Ershadi et al., 2021; Pushparaj et al., 2021; Wen et al., 2021; Su et al., 2024), and so warrants further investigation. Figure 10B depicts the chemical structure of TSA. The clustering result obtained from the L1000CDS^2^ analysis showed that for the uploaded COVID-19 gene signature, TSA may reverse the expression of serpin family H member 1 (SERPINH1), lysophosphatidic acid receptor 1 (LPAR1) and transcription elongation factor A2 (TCEA2). Furthermore, in the MDD gene signature, TSA may reverse the expression of nucleolar protein 3 (NOL3), LPAR1 and heat shock protein family A member 6 (HSPA6) (Figure 10C).

### Molecular docking and MD simulation analysis

**FIGURE 11:**
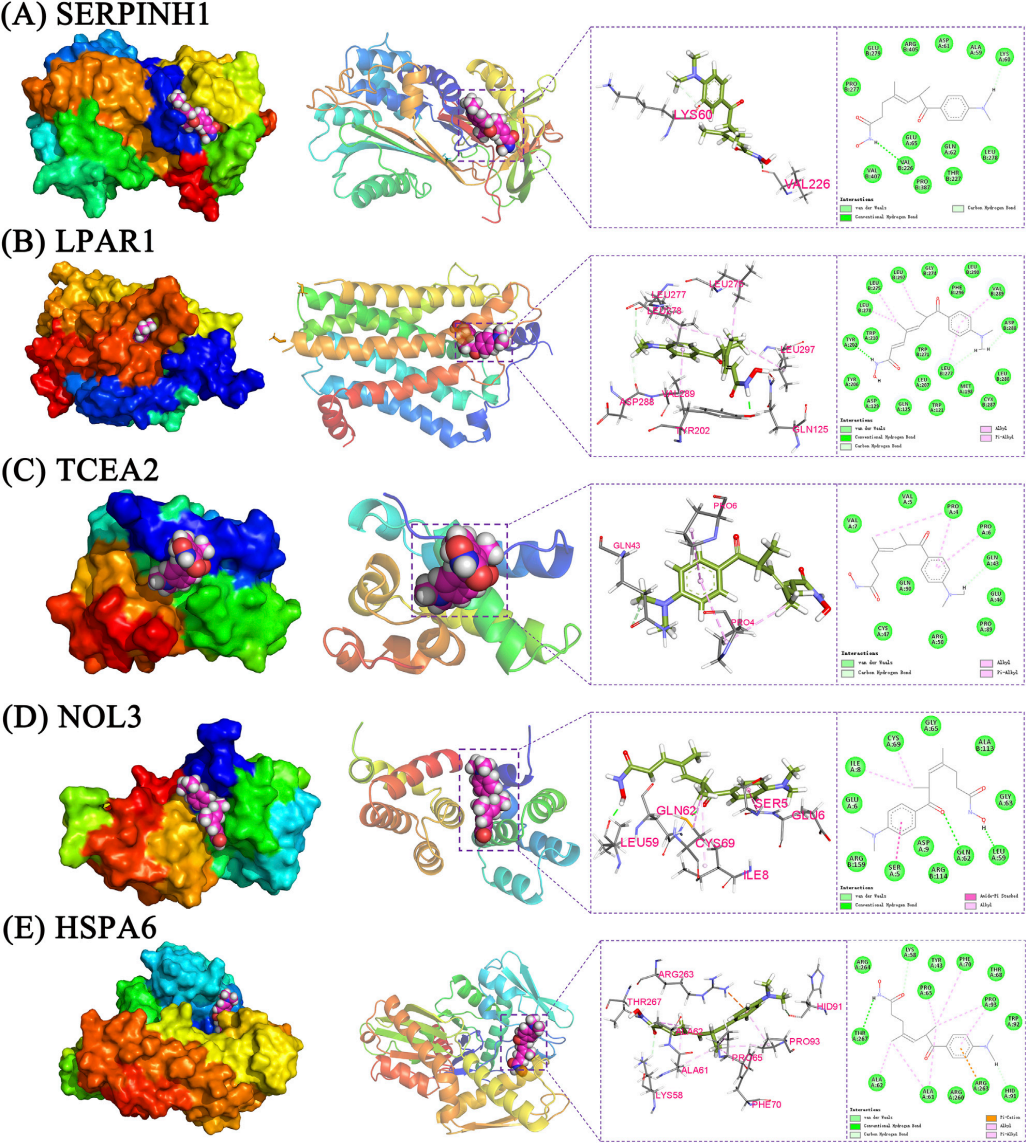
The interaction between TSA and proteins. **(A)** SERP1NH1; **(B)** LPAR1; **(C)** TCEA2; **(D)** NOL3; **(E)** HSPA6.

TSA, along with the perturbed proteins SERPINH1, LPAR1, TCEA2, NOL3, and HSPA6, were selected for molecular docking analysis. As listed in Table 1, the docking free energies of TSA with SERPINIH1, LPAR1, TCEA2, NOL3, and HSPA6, were −6.1, −7.6, −5.6, −6.2, and −7.3 kcal/mol, respectively. These values indicated the binding partners in the complex have a relatively strong binding affinity. In the TSA–SERPINIH1 complex, residue VAL226 forms a hydrogen bond, whereas ASP61, VAL407, and LEU278 interact through van der Waals forces (Figure 11A). In the TSA–LPAR1 complex, TYR202 establishes a hydrogen bond, whereas LEU297, LEU275, LEU278, VAL289, and LEU277 exhibit hydrophobic interactions characterized by alkyl and pi-alkyl contacts. Furthermore, ASP129, GLY274, and LEU280 engage in interactions via van der Waals forces (Figure 11B). In the TSA–TCEA2 complex, PRO4 and PRO6 display hydrophobic interactions via alkyl and pi-alkyl contacts, respectively. Meanwhile, VAL5, GLU46, and CYS47 undergo van der Waals interactions (Figure 11C). In the NOL3–TSA complex, residues LEU59 and GLN62 form hydrogen bonds. Additionally, SER5 exhibits hydrophobic amide-pi stacking interactions, while CYS69 and ILE8 display hydrophobic alkyl interactions. Furthermore, GLY65, ARG159, and GLY63 engage in van der Waals interactions (Figure 11D). In the HSPA6–TSA complex, THR267 forms a hydrogen bond. Furthermore, ARG263, ALA61, ALA62, PRO93, PRO65, and PHE70 exhibit hydrophobic pi-cation, alkyl, and pi-alkyl interactions. Additionally, ARG264, ARG260, and TRP92 engage in van der Waals interactions (Figure 11E).

**TABLE 1:** The docking energy (kcal/mol) of the TSA–protein complex.

Protein	PDB ID	Docking energy (kcal/mol)
SERPINH1	3ZHA	−6.1
LPAR1	4Z34	−7.6
TCEA2	2LW4	−5.6
NOL3	4UZ0	−6.2
HSPA6	3FE1	−7.3

**FIGURE 12:**
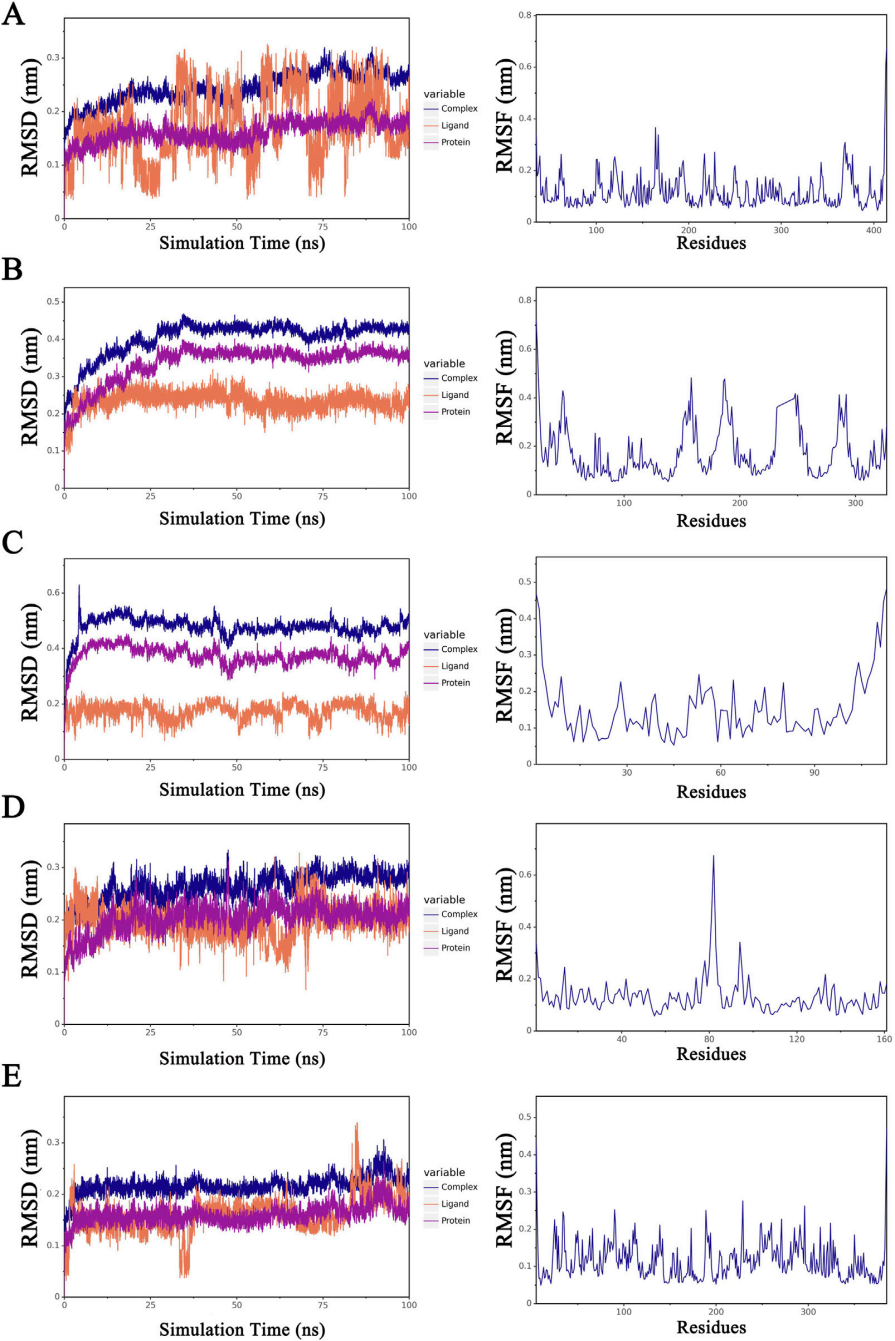
RMSD and RMSF values for the TSA–protein complex. **(A)** SERP1NH1; **(B)** LPAR1; **(C)** TCEA2; **(D)** NOL3; **(E)** HSPA6.

The root mean square deviation (RMSD) is a crucial metric for assessing the stability of protein–ligand interactions. As shown in Figure 12, the RMSDs of five proteins complexed with TSA, namely, SERPINH1, LPAR1, TCEA2, NOL3, and HSPA6, ranged between 0 and 0.5 nm, whereas the RMSD of the TCEA2–TSA complex had a wider range of 0–0.7 nm (Figure 12). Root mean square fluctuation (RMSF) is an indicator of the flexibility of amino acid residues within a protein, offering valuable insights into local variations in flexibility along the protein chain (Serseg et al., 2023). Figure 12 depicts the RMSF changes observed among the five proteins, with SERPINH1 and HSPA6 having narrower fluctuation ranges than the other proteins (Figure 12). The interaction energies corresponding to the TSA-protein complexes from MD simulations were presented in Table 2. The results of RMSD and RMSF analyses indicated that the structures of the TSA–protein complexes were highly stable.

**TABLE 2:** The binding energy (kcal/mol) of the TSA–protein complex in MD simulations.

Complex	ΔE_vdw_	ΔE_ele_	ΔE_pol_	ΔE_nonpol_	ΔE_MMPBSA_	-TΔS	ΔG_bind_*
HSPA6-TSA	−141.025 ± 5.782	−34.394 ± 9.446	109.3 ± 9.378	−21.101 ± 0.303	−87.22 ± 0.931	32.873 ± 2.774	−54.346 ± 1.963
LPAR1-TSA	−182.081 ± 1.889	−57.7 ± 3.336	167.138 ± 2.529	−23.289 ± 0.143	−95.932 ± 5.781	16.353 ± 4.254	−79.579 ± 8.592
NOL3-TSA	−131.517 ± 3.924	−23.05 ± 3.289	108.655 ± 6.795	−17.604 ± 0.272	−63.516 ± 0.459	23.402 ± 2.323	−40.114 ± 2.776
SERPIINH1-TSA	−82.484 ± 4.963	−5.15 ± 1.981	48.803 ± 5.353	−13.28 ± 0.415	−52.112 ± 2.849	23.74 ± 6.397	−28.371 ± 9.228
TCEA2-TAS	−104.208 ± 9.481	−11.144 ± 3.401	65.527 ± 11.961	−15.825 ± 1.39	−65.65 ± 3.259	26.728 ± 1.842	−38.922 ± 4.95

## Discussion

Inflammation, a common characteristic of nearly all diseases, likely contributes to COVID-19-related depressive symptoms (Lyra et al., 2022). Inflammation is closely linked to the activation of microglia, elevated levels of pro-inflammatory cytokines, excessive activity of the HPA axis, alternations in neuroplasticity, and changes in the makeup and diversity of gut microbiota (Yin et al., 2024). These factors, either individually or collectively, may contribute to the development of mental health disorders (Yin et al., 2024). Two significant cytokines elevated during COVID-19, TNF-α and IL-6, directly affect brain physiology and may drive dysfunctional stress-related responses, mood alternations, and depressive symptoms (Lyra et al., 2022). In 226 COVID-19 survivors from Milan, Italy, the persistence of depressive symptoms was linked to systemic inflammatory markers present during acute infection and during follow-up visits (Mazza et al., 2021). Compared with the latest research on transcriptomic analysis of peripheral blood neutrophils (Divolis et al., 2025), we found that 13.8% of DEGs identified in the cerebral cortex are also present in peripheral neutrophils (Supplementary Figure S1). This observation indicated the existence of commonly regulated immune response across different tissues in COVID-19.

Alternatively, COVID-19 and depression disorders may share a common genetic basis (Pan et al., 2021; Lyra et al., 2022; Yin et al., 2024). The current study uncovered 60 DEGs that were common to both COVID-19 and MDD (Figure 2). Moreover, these 60 common DEGs exhibited unique expression profiles in COVID-19 and depression (Figure 3).

Enrichment analysis offers a detailed way to understand the biological functions of gene sets (Zhao and Rhee, 2023; Chang et al., 2024). Our findings indicated that the primary biological processes associated with these 60 common DEGs are predominantly linked to protein folding. The top two biological processes were “response to unfolded protein” and “response to topologically incorrect protein” (Figure 4A). Protein folding is essential for the life cycle of viruses, including Sars-CoV-2, being involved in viral structure, pathogenesis, and the defense reaction of host cells (Keramidas et al., 2024). KEGG analysis revealed that the primary pathways involved were “MAPK signaling pathway”, “Legionellosis”, and “Transcriptional misregulation in cancer” (Figure 4B). These findings contribute to a more comprehensive understanding of the functions of the DEGs. MAPK activation is involved in neurotoxicity induced by the SARS-CoV-2 spike protein (Kyriakopoulos et al., 2022). Legionellosis is a type of pneumonia that can be acquired in the community and within healthcare settings (Chalker et al., 2021). Patients with SARS-CoV-2 might be at increased risk for other community- or healthcare-acquired infections, such as possible *Legionella pneumonphila* infection (Chalker et al., 2021). *Legionella* infection can affect the central nervous system, leading to neuro-cytotoxic effects (Sauchelli et al., 2012). These mechanistic insights will enhance our comprehension of the biological functions that underlie the development and progression of COVID-19-related depression.

Hub genes are those that interact with many other genes and are crucial to biological processes and gene regulation, and as such serve as a measurable indicator of the physiological or pathological condition of an organism (Wang et al., 2022). Machine learning and AI have already been successfully used in biomarker discovery (Ng et al., 2023). We used LASSO and RF methods to identify EMILIN3, OPA3, and TFCP2, as potential hub genes for both COVID-19 and MDD (Figure 5). Furthermore, OPA3 and TFCP2 are found in the DEGs expressed in peripheral blood neutrophils of COVID-19 patients (Supplementary Figure S1).

EMILIN3 belongs to a family of secreted glycoproteins characterized by a distinctive N-terminal cysteine-rich EMI domain (Schiavinato et al., 2012). EMILIN3 is an antagonist of transforming growth factor β (TGF-β) (Schiavinato et al., 2012). The EMILIN3 gene augments the aggressiveness of low-grade gliomas, a prevalent type of brain tumor (Wang L. A. et al., 2024). Furthermore, OPA3, a protein located in the mitochondrial membrane, is a novel regulator of lipid metabolism (Wells et al., 2012). Mutations in the OPA3 gene are commonly associated with optic neuropathy and the formation of cataracts (Sergouniotis et al., 2015; Bagli et al., 2017). Moreover, TFCP2 is a globin transcription factor that plays crucial roles in various human conditions, including cancer, Alzheimer’s disease, embryonic development, blood pressure regulation, and brain function (Taracha et al., 2018). It appears to be a significant factor in MDD pathogenesis (Taracha et al., 2018). TFCP2 can influence the risk of developing both MDD and Alzheimer’s disease (Schahab et al., 2006). Based on PubMed literature searches (conducted in September 2024), there are limited reports on the three hub genes in the context of COVID-19 and MDD and so their potential roles in COVID-19-related depression remain to be further explored.

Distinct gene expression patterns, or gene signatures, can be used to identify disease subtypes, drug candidates and drug targets, and to elucidate the underlying biological mechanisms (Lamb et al., 2006; Imami et al., 2021). Gene signatures are frequently used in research to identify drug candidates for COVID-19 (Imami et al., 2021; Karami et al., 2021). In this study, L1000CDS^2^ predicted the top 36 small-molecule compounds that could reverse the gene-expression signature associated with COVID-19 (Figure 10A; Supplementary Table S4). Furthermore, the analysis also identified the top 38 small-molecule compounds for MDD (Figure 10A; Supplementary Table S5). Notably, TSA, vorinostat, perhexiline maleate, PI 103 hydrochloride, quabain, and digoxin emerged as common drug candidates for both COVID-19 and MDD (Figure 9A).

Vorinostat, a histone deacetylase (HADC) inhibitor, is approved for treating cutaneous T-cell lymphoma (https://go.drugbank.com/drugs/DB02546). It has reported antidepressant effects (Covington et al., 2009; Kv et al., 2018; Misztak et al., 2021; Nasehi et al., 2022). Vorinostat alleviates inflammatory damage and oxidative stress in the chronic corticosterone-induced stress model (Kv et al., 2018; Misztak et al., 2021). Additionally, it improves the symptoms of depression comorbid with cardiovascular diseases (Nasehi et al., 2022), and reduces depression-like behavior during ethanol withdrawal [42]. The antidepressant mechanisms of vorinostat may involve alterations in acetylated histone H3 levels and HDAC2 expression, mediating long-lasting positive neuronal adaptations (Covington et al., 2009). However, in the context of COVID-19, vorinostat increases SARS-CoV-2 RNA abundance, augments virus infection in cell models (Ravindran et al., 2022), and upregulates the ACE2 receptor that facilitates SARS-CoV-2 cell entry (Sinha et al., 2020). Therefore, vorinostat exhibits a clear pro-viral effect, rendering it unsuitable for treating depression in the context of COVID-19.

Both ouabain and digoxin are plant-derived cardiac glycoside drugs that enhance cardiac function by inhibiting the Na + -K + -ATP enzyme on the myocardial cell membrane, thereby strengthening myocardial contractility (Patel, 2016). Digoxin or ouabain effectively inhibit SARS-CoV-2 replication during the post-entry stage of the viral life cycle (Cho et al., 2020). In addition, ouabain induces mania-like behavior and depressive-like behavior in rats, and increases inflammatory markers, such as interleukin (IL)-1β, IL-6, IL-10, TNF-α, and CINC-1 in the frontal cortex and hippocampus of rats (Valvassori et al., 2022). Moreover, both ouabain and digoxin exhibit a very narrow therapeutic index, which limits their application in severely ill patients (Ahmed et al., 2023). Additionally, a search of the Pubmed database (conducted in September, 2024), identified only a limited number of research articles on the relationship between perhexiline maleate and PI 103 hydrochloride in the context of COVID-19 or depression. Therefore, these compounds, i.e., vorinostat, ouabain, digoxin, perhexiline maleate and PI 103 hydrochloride, are likely unsuitable candidates for treating COVID-19-related depression.

TSA is a potential small molecule compound predicted by L1000CDS^2^ to act on the disease expression profiles of both COVID-19 and depression simultaneously (Figure 10A). TSA (Figure 10B) is a hydroxamic acid originally derived from the secondary metabolites of *Streptomyces hygroscopicus* strains (Tsuji et al., 1976). It is effective in suppressing SARS-CoV-2 main protease activity and SARS-Cov-2 replication *in vitro*, disrupting the post-entry events of the SARS-CoV-2 replication cycle (Wen et al., 2021). Furthermore, TSA can reverse gene signatures related to long-term neurologic outcomes of COVID-19 (Pushparaj et al., 2021). In addition, TSA alleviates depression-like behavior in APP/PS1 mice in the forced swimming test, possibly due to its inhibition of CST7-related microglial inflammation (Su et al., 2024). Similarly, in mice, TSA attenuates depressive-like behavior, cognitive function and inflammatory response in male offspring subjected to maternal separation (Ershadi et al., 2021). Moreover, TSA alleviated depression-like behaviors and restored normal epigenetic patterns in the hippocampus during ethanol withdrawal in rodent models (Chen et al., 2019). Therefore, TSA might serve as a promising drug candidate for treating COVID-19-related depression, which is worthy of further research.

The half-maximal effective concentration (EC50) of TSA for inhibiting SARS-CoV-2 replication ranged from 1.5 to 2.7 µM, which is notably lower than its 50% cytotoxic concentration (75.7 µM) and peak serum concentration (132 µM) (Wen et al., 2021). Furthermore, due to TSA’s extremely short plasma half-life, it is essential to conduct further optimization of the drug compound to develop more stable analogs that possess extended half-livers (Wen et al., 2021). This information will further facilitate the drug development of TSA.

Moreover, the L1000CDS^2^ clustergram results revealed an overlap in gene expression between the input genes and those perturbed by TSA. Overlapping genes for TSA and COVID-19 were SERPINH1, LPAR1, and TCEA2 (Figure 10C), and overlapping genes for TSA and MDD were NOL3, LPAR1, and HSPA6. Notably, LPAR1 appeared in both results (Figure 10C). Additionally, a Sankey diagram showed that LPAR1 participates in the “Phospholipase D signaling pathway” (Figure 4C).

SERPINH1 (also known as Hsp47) and HSPA6 both belong to the HSP family (Widmer et al., 2012; Song et al., 2022). SERPINH1 facilitates the accurate assembly of triple-helical procollagen molecules (Widmer et al., 2012). HSPA6 is highly expressed in the cerebral cortex (Song et al., 2022). HSP proteins are involved in tumorigenesis and progression, as well as various other diseases, and are potential drug targets for treating a wide range of conditions (Widmer et al., 2012; Song et al., 2022). Additionally, in COVID-19 patients with a ‘profibrotic phenotype’, HSP47 is detected in myofibroblast clusters, suggesting its role in facilitating extracellular matrix (ECM) remodeling and cardiac fibrosis during SARS-CoV-2 infection (Jacobs et al., 2023). HSP70 and HSPA6 exhibited highly expression level in cells infected with human coronaviruses and demonstrate the ability of evading the virus-induced translational blockade (Pauciullo et al., 2024). Furthermore, HSP 70 has been identified as a potential biomarker for depression (Sun et al., 2018). These suggests that HSP47 and HSPA6 might be involved in the pathogenesis of COVID-19 depression.

Lysophosphatidic acid (LPA) is a pleiotropic bioactive lipid molecule that signals through LPAR1 (Chrencik et al., 2015). The binding pocket of LPAR1 for the indane moiety of a tool antagonist is constituted by GLY274 and Leu275 (Chrencik et al., 2015). TSA can form hydrophobic interactions with the residue LEU275 and van der Waals force interactions with GLY274 (Figure 11B). The interaction between LPA and its receptor LPAR1, activates ROCK1/2 kinases, which in turn suppresses of IFN I/III production and consequently hindering viral clearance (Zhang et al., 2021). Notably, LPAR1 has emerged as a novel therapeutic target for depression and a specific new target of antidepressant interventions (Kajitani et al., 2016; Kajitani et al., 2024). These also indicates that LPAR1 may be implicated in the mechanism of COVID-19-related depression.

NOL3 is an apoptosis repressor that contains a caspase recruitment domain [60]. TCEA2 encodes a protein that is primarily situated within the nucleus and functions as an SII-type transcription elongation factor (Zhao et al., 2024). Our molecular docking and MD results support the notion that complexes of TSA with either one of the five proteins exist in a stable state (Figure 12).

Notably, a research report on COVID-19-related depression published while we were conducting the current study utilizes the same datasets as our study, GSE188847 for COVID-19 and GSE101521 for MDD (Zhuang et al., 2024). However, our approaches are different to that study in several key aspects, namely, sample selection, and methods used to identify hub genes and predict drugs that perturb gene signatures. Specifically, for MDD samples we used data from 29 controls and 9 MDD patients who did not die by suicide, whereas the other report included these patients, as well as 21 patients who died by suicide (Zhuang et al., 2024). Furthermore, the experimental methods used differ significantly. We chose two machine learning approaches, whereas they utilized the weighted gene co-expression network analysis (WGCNA) (Zhuang et al., 2024). Consequently, the research pinpointed four key genes, MBP, CYP4B1, ERMN, and SLC26A7, which hold clinical relevance and exhibit potential significance in the pathophysiology of COVID-19 induced depression (Zhuang et al., 2024). While there are certain variations in the experimental results, both studies offer valuable insights into the mechanisms underlying COVID-19-related depression.

## Summary

The principal goal of this study was to evaluate the gene expression profile, identify key genes, and discover potential therapeutic agents for the co-occurrence of COVID-19 and MDD. We identified 60 DEGs that overlapped yet exhibited distinct expression patterns in the COVID-19 (GSE188847) and MDD (GSE101521) datasets. Subsequently, two machine learning analyses, LASSO and RF, revealed that EMILIN3, OPA3, and TFCP2 as potential hub genes. Furthermore, an L1000CDS^2^ analysis of the gene-expression signatures indicated that TSA, a metabolite derived from *Streptomyces*, could reverse the altered gene expression and potentially serve as a therapeutic option for COVID-19-associated depression. Moreover, molecular docking and MD simulation results revealed that the TSA–perturbed protein complexes form spontaneously and are relatively stable. In summary, TSA is a promising candidate for the clinical treatment of COVID-19-related depression. However, given the inherent limitations of bioinformatics research, supplementary wet-lab investigations are needed to validate these discoveries.

## Data Availability

Publicly available datasets were analyzed in this study. This data can be found here: The GEO database provides open access to the raw data for GSE188847 and GSE101521.

## Supplementary material

SUPPLEMENTARY TABLE S1 DEGs for COVID-19 (GSE188847).

SUPPLEMENTARY TABLE S2 DEGs for MDD (GSE101521).

SUPPLEMENTARY TABLE S3 The 60 identified DEGs for both COVID-19 and MDD.

SUPPLEMENTARY TABLE S4 The top 50 potential drug candidates for COVID-19.

SUPPLEMENTARY TABLE S5 The top 50 potential drug candidates for MDD.

SUPPLEMENTARY FIGURE S1 Overlapping DEGs between brain cortex and peripheral blood neutrophils in COVID-19 patients. The data for peripheral blood neutrophils are from the latest research (Divolis et al., 2025).
