# Supplementary figures and images for: Identification of gene signatures and potential pharmaceutical candidates linked to COVID-19-related depression based on gene expression profiles
Source: Front Pharmacol. 2025 Aug 22;16:1562774. doi: 10.3389/fphar.2025.1562774 (PMC12411777; doi:10.3389/fphar.2025.1562774)

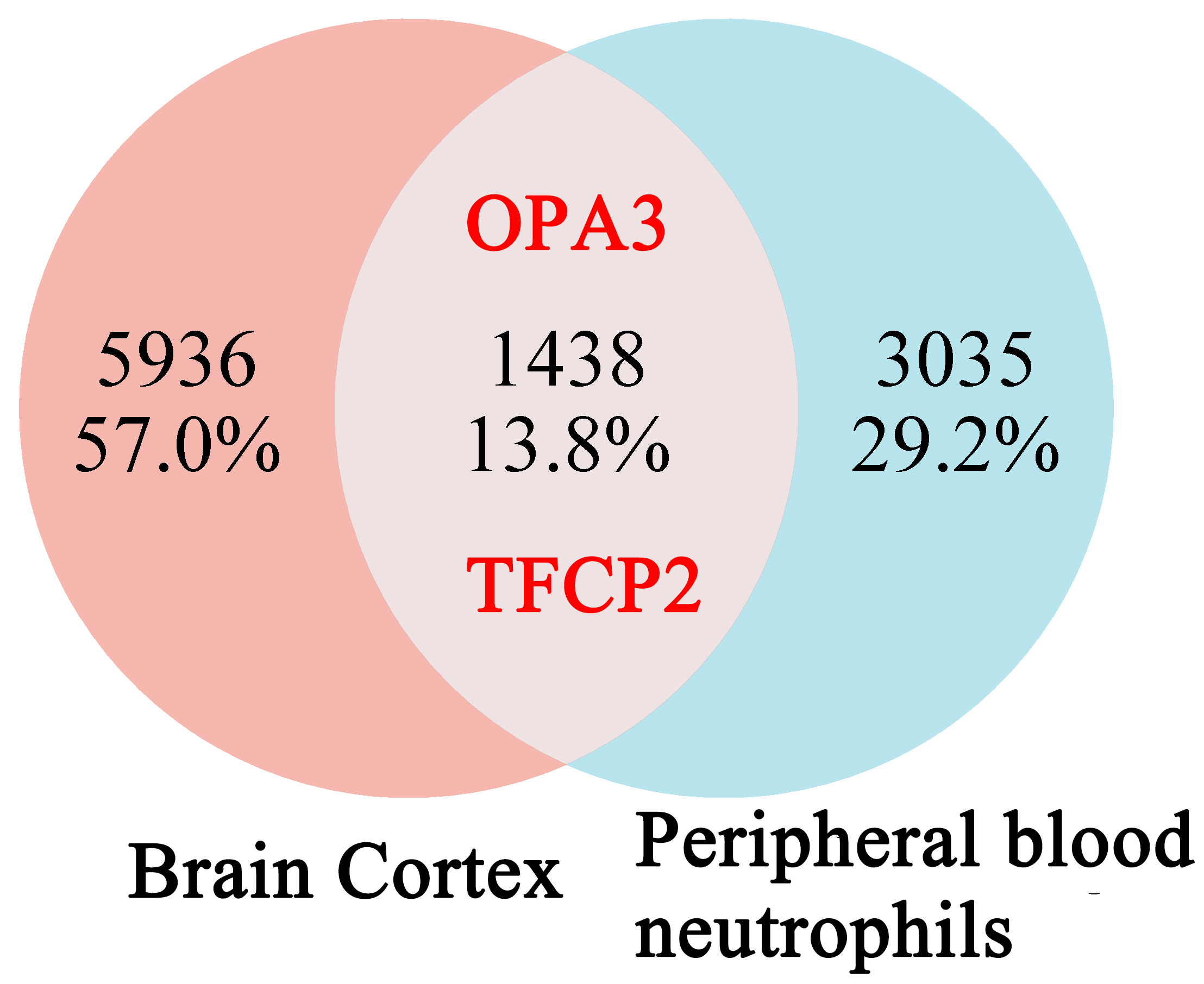

Supplement: Supplementary file 2 [file Image1.tif]
